# Association of *Porphyromonas gingivalis* with Acute Myocardial Infarction: A Systematic Review

**DOI:** 10.3390/jcm15145689

**Published:** 2026-07-20

**Authors:** Elina Ghondaghsaz, Edward E. Putnins, Ahmed Hieawy

**Affiliations:** 1 Department of Neuroscience, University of British Columbia, Vancouver, BC V6T 1Z3, Canada; elinagho@student.ubc.ca; 2 Department of Oral Biological and Medical Sciences, Faculty of Dentistry, University of British Columbia, Vancouver, BC V6T 1Z3, Canada; ed.putnins@ubc.ca; 3 Vancouver Coastal Health Research Institute, Vancouver General Hospital, Vancouver, BC V5Z 1M9, Canada; 4 BC Children’s Hospital Research Institute, BC Children’s Hospital, Vancouver, BC V5Z 4H4, Canada

**Keywords:** *Porphyromonas gingivalis*, myocardial infarction, periodontitis, cardiovascular diseases, systematic review, virulence

## Abstract

**Background/Objectives:** Periodontitis is a chronic oral infection in which *Porphyromonas gingivalis* (*Pg*) acts as a keystone pathogen capable of systemic dissemination and immune evasion. A possible association between *Pg* and acute myocardial infarction (AMI) has been proposed; this systematic review is, to our knowledge, among the first to evaluate this association through a pathogen-specific synthesis integrating both microbial and serological evidence. **Methods:** Five electronic databases (PubMed, Scopus, Embase, Web of Science, Cochrane Library) were searched from inception to February 2025, with a supplementary top-up search performed in July 2026 that identified no additional eligible studies. Observational studies assessing *Pg* presence or anti-*Pg* antibody levels in participants with and without AMI were eligible. Methodological quality was assessed using the Newcastle–Ottawa Scale (NOS). Findings were synthesised narratively due to substantial clinical and methodological heterogeneity. The protocol was registered in PROSPERO (CRD42025644043). **Results:** Twelve case-control studies (5147 participants; 2518 AMI cases, 2629 controls) were included. Six evaluated serum anti-*Pg* antibodies and six used direct microbial or molecular detection. Three studies in each category reported a significant *Pg*–AMI association; three in each category did not. NOS scores ranged from 6 to 9 (eight studies rated good quality; four fair quality). Heterogeneity in antigen selection, sampling site, immunoglobulin isotype, and confounder adjustment precluded meta-analysis. **Conclusions:** The available evidence suggests a possible but inconsistent association between *Pg* and AMI, insufficient to establish a causal relationship. Standardised detection protocols and prospective longitudinal studies with comprehensive confounder adjustment are needed. The detection of *Pg* in isolation is not currently validated for AMI risk stratification, and the available evidence does not establish a causal relationship between *Pg* and AMI.

## 1. Introduction

Acute myocardial infarction (AMI) typically results from the rupture of a vulnerable atherosclerotic plaque and the consequent thrombotic occlusion of a coronary artery [1]. Coronary artery disease is the principal underlying condition and remains the leading cause of death worldwide [2]. Well-established modifiable risk factors for AMI include hypertension, dyslipidaemia, diabetes mellitus, tobacco use, physical inactivity, and obesity [3,4]. Evidence from large longitudinal cohorts suggests that the majority of AMI events could be prevented through sustained lifestyle modification and pharmacological risk factor control [5,6]. Beyond conventional risk factors, systemic infections have increasingly been recognised as contributors to AMI risk. A large Danish cohort study spanning three decades found that common bacterial infections were associated with significantly elevated AMI rates, with inflammatory and procoagulant mechanisms proposed as the underlying pathways [7]. Autopsy and experimental data further indicate that systemic infection can destabilise existing atheromatous plaques through heightened inflammatory activity and oxidative stress [8,9].

Periodontitis is a chronic inflammatory disease of the tooth-supporting structures and one of the most prevalent oral infectious diseases globally [10]. Global burden-of-disease estimates indicate that severe periodontitis affects approximately 11% of adults worldwide, making it the sixth most prevalent condition globally, with prevalence rising markedly with age [11]. *Porphyromonas gingivalis* (*Pg*), a Gram-negative anaerobic bacterium, is regarded as a keystone pathogen in periodontitis owing to its capacity to subvert innate immune responses and disseminate systemically via bacteraemia [12,13,14]. It frequently co-exists with *Treponema denticola* and *Tannerella forsythia*—collectively termed the red complex—whose synergistic interactions amplify local tissue destruction and systemic inflammatory burden [15,16,17]. Serum immunoglobulin responses against *Pg* have been proposed as a surrogate marker of periodontal disease severity and systemic pathogen burden [18,19,20,21,22].

Mechanistically, *Pg* possesses a broad repertoire of virulence factors, including fimbriae, capsule, arginine- and lysine-specific gingipain proteases, outer membrane vesicles, and atypical lipopolysaccharide, which collectively facilitate immune evasion, complement dysregulation, tissue invasion, and transient bacteraemia [13,14,23]. Following entry into the bloodstream, *Pg* has been shown to invade vascular endothelial cells, vascular smooth muscle cells, and macrophages, where it contributes to persistent vascular inflammation. In addition, *Pg* DNA has been detected within atherosclerotic plaques, supporting its presence in vascular lesions and providing biological plausibility for a contributory role in atherogenesis and plaque instability [24,25]. Experimental studies further suggest that *Pg* promotes macrophage foam-cell formation through enhanced oxidised low-density lipoprotein uptake and impairs cardiomyocyte autophagy following myocardial infarction, mechanisms that could contribute to the development and progression of acute coronary events [26,27]. Collectively, these mechanistic findings provide biological plausibility for the epidemiological associations between *Pg* exposure and acute myocardial infarction examined in this review.

Several meta-analyses have examined the association between periodontitis and cardiovascular outcomes. Qin et al. reported a 13% higher relative risk of AMI in patients with periodontal disease across ten cohort studies, though without a pathogen-specific subgroup analysis [23]. Joshi et al. conducted a meta-analysis of 20 studies in coronary artery disease patients, reporting a pooled odds ratio of 1.23 (95% CI 1.09–1.38) for anti-*Pg* immunoglobulin G (IgG), though these studies broadly sampled coronary artery disease rather than AMI specifically [24]. Larvin et al. and Leng et al. confirmed elevated cardiovascular disease risk in periodontitis patients across large cohorts [25,26]. To date, no systematic review has focused specifically on the association between *Pg*—assessed by direct microbial detection or anti-*Pg* antibody measurement—and the occurrence of AMI. Such specificity is warranted given the unique virulence profile of *Pg* [13,27]. Antibody-based and microbial detection methods capture complementary aspects of *Pg* exposure: serum anti-*Pg* antibody titres reflect cumulative or chronic host exposure to *Pg* antigens over time, whereas direct microbial or molecular detection in subgingival plaque or blood reflects current bacterial presence or bacteraemic dissemination [18,19,20,21,22]. This review deliberately incorporated both serological and microbial evidence to capture complementary dimensions of *Pg* exposure by integrating markers of cumulative host immune response with evidence of current bacterial detection, both of which may be relevant to AMI risk. The present systematic review aims to evaluate the evidence linking *Pg* exposure to AMI risk, enabling a more pathogen-specific analysis than has previously been undertaken.

## 2. Materials and Methods

### 2.1. Protocol and Reporting

This systematic review was designed and reported in accordance with the Preferred Reporting Items for Systematic Reviews and Meta-Analyses (PRISMA) 2020 guidelines [28]. The completed PRISMA checklist is provided in Supplementary Table S1. The review protocol was prospectively registered in PROSPERO (registration number CRD42025644043) on 7 February 2025, prior to the commencement of title/abstract screening and data extraction.

### 2.2. Search Strategy

A comprehensive literature search was conducted across five international databases—PubMed, Scopus, Embase, Web of Science, and the Cochrane Library—from inception to February 2025. Search terms were organised into two categories: Category A (myocardial infarction and related cardiovascular terms) and Category B (*Pg* and synonyms), combined using the Boolean operator AND. Full search strings are provided in Supplementary Table S2. Grey literature were not searched; this is acknowledged as a potential source of publication bias. A supplementary top-up search using the same terms was performed in July 2026 to identify any eligible studies published between February 2025 and submission. This identified no additional clinical studies meeting the review’s inclusion criteria; the studies published in this interval were mechanistic or *in vitro* investigations of *Pg*–atherosclerosis pathways rather than clinical studies of *Pg* exposure and AMI outcome. The evidence base underlying this review’s conclusions is therefore considered current as of submission.

### 2.3. Eligibility Criteria

Inclusion criteria: (1) observational studies or randomised controlled trials assessing *Pg* presence or anti-*Pg* antibody levels in participants with and without AMI; (2) human subjects of any age or sex; (3) clearly defined diagnostic criteria for both *Pg* exposure and AMI; and (4) English-language publications. The restriction to English-language publications was applied to allow accurate extraction of diagnostic and outcome data without reliance on translation; however, it is acknowledged that this restriction may have excluded relevant non-English studies and introduced potential language bias, which is discussed further as a limitation.

Exclusion criteria: (1) preclinical studies (*in vivo* or *in vitro*); (2) cardiovascular outcomes other than AMI unless AMI data were extractable; (3) narrative reviews, case reports; (4) duplicate studies; (5) incomplete outcome data; and (6) conference abstracts or preprints.

### 2.4. Study Selection

**Figure 1:**
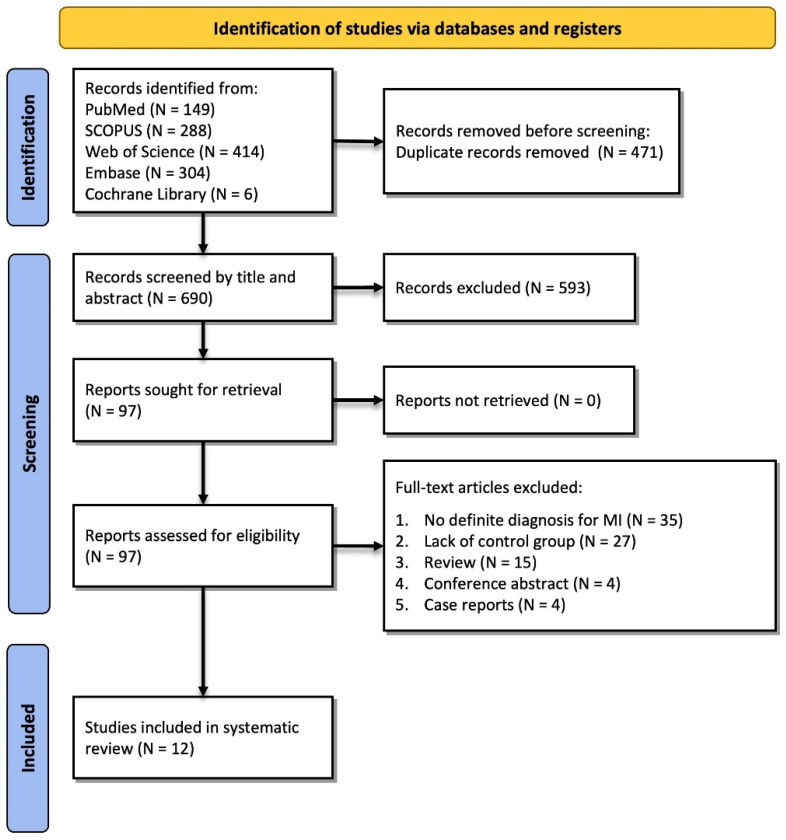
Study selection flow diagram (PRISMA 2020) [28]. From 1161 records retrieved across five databases (13 February 2025), 690 titles and abstracts were screened, and 12 studies met the inclusion criteria following full-text review of 97 articles.

Records were imported into EndNote 20 and deduplicated. Two reviewers (E.G. and A.H.) independently screened titles and abstracts, followed by full-text assessment of potentially eligible studies. Disagreements were resolved by consensus; where consensus could not be reached, a third reviewer was consulted. Eligibility was structured around a PECO framework (Population: individuals with and without AMI; Exposure: *Pg* presence or anti-*Pg* antibody level; Comparator: AMI-free controls; Outcome: occurrence of AMI). Screening was performed manually without automation or machine learning-assisted tools. The reference lists of all included studies were manually screened for additional eligible records; no further studies were identified through this process. Inter-rater agreement was quantified using Cohen’s kappa (k = 0.82), indicating strong agreement. The study selection process is summarized in the PRISMA flow diagram (Figure 1).

### 2.5. Data Extraction

Two reviewers independently extracted data covering: (1) study characteristics; (2) *Pg* detection method; (3) AMI diagnostic criteria; (4) primary outcomes; and (5) adjusted confounders. For studies reporting medians and interquartile ranges, conversion to means and standard deviations followed the methods of Luo et al. and Wan et al. [29,30]. Discrepancies were resolved by consensus. Data extraction used a standardised, piloted extraction form capturing the exact wording of each study’s AMI diagnostic definition, the effect estimates and measures of variability reported (odds ratios, relative risks, or *p*-values, as available), and the specific confounders entered into each adjusted model, to support the narrative and comparative synthesis presented in Section 3.

### 2.6. Quality Assessment

Methodological quality was assessed using the Newcastle–Ottawa Scale (NOS) for case-control studies, evaluating selection (maximum 4 stars), comparability (maximum 2 stars), and exposure (maximum 3 stars), yielding a maximum total of 9 stars. Studies scoring 7–9 were rated good quality, 5–6 fair quality, and 0–4 poor quality. Two reviewers assessed each study independently. Across studies, comparability stars were most often lost due to non-adjustment for smoking or diabetes status, and exposure stars were most often lost where *Pg* status was ascertained by self-report rather than direct clinical or laboratory verification. A formal GRADE assessment was not performed. Although GRADE can be applied without a pooled estimate, we judged this impractical given the marked heterogeneity in AMI definitions and *Pg* exposure metrics across studies (Section 3.5), which limits a coherent summary judgement; this is noted as a limitation. In addition, since the NOS assesses risk of bias at the study level rather than the outcome level, and several studies reported multiple *Pg*-related exposure metrics (e.g., IgG/IgA, or oral versus blood detection), residual outcome-level bias (particularly selective outcome reporting) cannot be ruled out and is also acknowledged as a limitation.

### 2.7. Data Synthesis

Meta-analysis was not feasible due to substantial clinical and methodological heterogeneity, including variation in *Pg* detection platforms, exposure types (antibody-based vs. direct microbial detection), outcome definitions, and population characteristics. Specifically, meta-analysis was precluded by a combination of inconsistent exposure definitions (antibody isotype and antigen target differed across studies), non-comparable effect metrics (several studies reported only *p*-values or descriptive comparisons without an extractable odds ratio, relative risk, or standard error), and heterogeneous outcome ascertainment (AMI defined variably across studies; see Section 3.5). As a complementary approach, structured vote-counting by direction of effect was considered; however, given the small number of studies (*n* = 12) and the fact that several reported multiple, partly overlapping exposure metrics within the same cohort, this was judged unlikely to add meaningfully to the narrative synthesis and risked overstating precision. This decision is discussed further as a limitation. Findings were therefore synthesised narratively. No formal publication bias assessment was conducted owing to the small number of included studies (*n* = 12) and the absence of a pooled statistical estimate; this is acknowledged as a limitation.

Where multiple publications originated from the same cohort, studies were retained separately in the qualitative synthesis when they reported distinct outcomes or analyses; however, participant numbers were counted only once when calculating the overall sample size to avoid double-counting.

## 3. Results

### 3.1. Study Selection Results

The systematic search retrieved 1161 records: 149 from PubMed, 288 from Scopus, 414 from Web of Science, 304 from Embase, and 6 from the Cochrane Library. After the removal of 471 duplicates, 690 records were screened, and 97 full-text articles were assessed for eligibility. Reasons for exclusion included: no definitive AMI diagnosis (*n* = 35), no control group (*n* = 27), and being a review article (*n* = 15), conference abstract (*n* = 4), or case report (*n* = 4). Twelve studies were retained for synthesis [12,31,32,33,34,35,36,37,38,39,40,41]. The PRISMA flow diagram is presented as Figure 1. Because the included studies varied in how AMI was ascertained, the specific diagnostic criteria used by each study (e.g., cardiac biomarkers, ECG findings, angiography, clinical records, or ICD coding) are summarised in Section 3.5 to facilitate the interpretation of differences in AMI outcome ascertainment across the included studies.

### 3.2. Baseline Characteristics of Included Studies

All 12 included studies used a case-control design; no cohort studies or randomised controlled trials met the inclusion criteria. A total of 5147 participants were enrolled (2518 AMI cases; 2629 controls). Two studies by Lund Haheim et al. [34,35] were derived from the same longitudinal cohort and were retained because they reported different analyses and outcomes. To avoid double-counting, participants from this cohort were included only once in the calculation of the overall sample size. Mean participant age ranged from 51.9 to 70.8 years. The proportion of male participants ranged from 53.1% to 100%. All studies were published between 2004 and 2023. Study characteristics are presented in Table 1.

Six studies employed serological analysis, quantifying serum anti-*Pg* IgG and/or immunoglobulin A (IgA) by enzyme-linked immunosorbent assay (ELISA), with heterogeneous antigen selection ranging from *Pg* arginine gingipain (Rgp) to a single reference strain (ATCC 3277) or multiple *Pg* serotypes. Six studies assessed *Pg* directly in biological samples using polymerase chain reaction (PCR), indirect immunofluorescence, dot-blot DNA hybridisation, or bacterial culture, primarily from subgingival plaque. One study additionally analysed circulating cell-free DNA (cfDNA) and genomic DNA (gDNA) in peripheral blood. Periodontal disease (PD) status was formally assessed in eight of the 12 studies.

### 3.3. Quality Assessment of Included Studies

NOS scores for all 12 studies are presented in Table 2. Eight studies were rated good quality (scores 7–9) and four were rated fair quality (scores 5–6). No study met the criteria for poor quality (scores 0–4). All 12 studies were retained for narrative synthesis. Discordance in findings was present even among studies achieving the highest NOS scores (score = 9), indicating that inconsistency reflects genuine biological and methodological heterogeneity rather than poor study design alone.

### 3.4. Association Between Pg and AMI

Where reported, effect estimates (odds ratios with 95% confidence intervals) are presented in Table 3 alongside the narrative summary below; however, most included studies reported only *p*-values or descriptive comparisons without an extractable odds ratio, relative risk, or standard error, which is itself part of the reporting heterogeneity underlying the decision not to pool data (Section 2.7).

#### 3.4.1. Serological Studies-Serum Antibody Analysis

Six studies compared anti-*PgPg* antibody levels between AMI cases and controls using ELISA [12,32,34,35,36,37]. Three reported a statistically significant association; three found no significant association in Table 3.

de Vries et al. [32] used *Pg* arginine gingipain as the antigen in 779 AMI cases and 719 healthy controls. Anti-Rgp IgG was significantly elevated in the AMI group overall (*p* = 0.035); however, this difference was non-significant when restricted to participants with confirmed periodontitis (*p* > 0.05). Lysek et al. [36] reported comparable median anti-*PgPg* gingipain IgG between 97 AMI cases and 113 controls (*p* = 0.36); a subgroup analysis identified that a moderate antibody titre was associated with approximately threefold higher odds of past AMI (odds ratio [OR] = 2.82, 95% confidence interval [CI] 1.02–7.84).

Holmlund et al. [12] reported elevated anti-*Pg* IgG in 100 AMI cases compared with 101 controls (*p* = 0.043), alongside higher periodontal pathogen counts; IgA levels were comparable between groups. Conversely, Pussinen et al. [37] found no significant IgG difference between 63 AMI cases and 63 controls (*p* = 0.876) but detected a significantly higher IgA in AMI patients (*p* = 0.035). Lund Haheim et al. published two analyses from the same longitudinal male cohort [34,35]. Anti-*Pg* IgG alone was non-significant in both analyses; however, a combined four-pathogen antibody panel was independently associated with AMI risk in the 2008 paper [34].

#### 3.4.2. Microbial Detection Studies-Plaque and Blood Samples

Six studies assessed *Pg* using direct microbial or molecular detection methods [31,33,38,39,40,41]. Three reported a significant positive association with AMI; three did not.

Stein et al. [40] reported the strongest positive association in this review, identifying *Pg* as an independent predictor of AMI after full adjustment for age, sex, smoking, body mass index, hypertension, plaque index, statin use, and cholesterol/high-density lipoprotein (HDL) ratio (OR = 13.6, 95% CI 3.1–59.8, *p* = 0.0005). Wu et al. [41] found oral swab PCR positivity was non-significant between 382 AMI cases and 78 controls, whereas circulating *Pg* cfDNA and gDNA in peripheral blood were significantly more frequent in AMI patients (*p* < 0.05 each), correlating with coronary artery disease (CAD) lesion severity. Pasupuleti et al. [38] reported significantly higher *Pg* load in AMI with generalised chronic periodontitis (GCP) compared with AMI alone (*p* = 0.043), suggesting the association may be contingent on concurrent PD.

Andriankaja et al. [31] found comparable *Pg* prevalence between 313 non-fatal AMI cases and 747 controls (18.5% vs. 15.8%; *p* > 0.05), with no independent association in adjusted models. *Tannerella forsythensis* and *Prevotella intermedia* independently predicted AMI in the same study, and an increasing number of co-detected pathogens elevated risk progressively. Dogan et al. [33] reported a significantly lower mean proportion of *PgPg* in patients with GCP and AMI compared with GCP alone (*p* = 0.05), suggesting an inverse rather than positive relationship. Seoane et al. [39] found *Pg* detection was more strongly associated with PD status than with AMI; no independent *Pg*–AMI association was identified after accounting for periodontal status.

### 3.5. AMI Diagnostic Criteria Across Included Studies

To facilitate the interpretation of the included evidence, Table 4 summarizes the myocardial infarction outcome label and diagnostic criteria reported in each study, including the methods used for outcome ascertainment (e.g., cardiac biomarkers, electrocardiographic findings, coronary angiography, clinical records, or ICD coding). Because the included studies varied in their definitions and ascertainment of myocardial infarction, the table also provides comments relevant to the review eligibility criteria and highlights sources of clinical heterogeneity that should be considered when interpreting the findings.

When the included studies are considered according to the certainty of AMI ascertainment (Table 4), seven studies diagnosed clinically confirmed acute myocardial infarction using cardiac biomarkers, electrocardiographic findings, and/or coronary angiography [12,32,33,38,39,40,41]. The remaining five did not meet this strict definition and instead used broader ascertainment: self-reported history of myocardial infarction [34,35], previously documented myocardial infarction obtained from medical records [36], and registry-identified incident myocardial infarction [37]; in one of the self-reported studies, cardiovascular mortality was the primary outcome [35]. A further study [31] diagnosed confirmed non-fatal acute myocardial infarction by WHO criteria but was restricted to survivors. When interpretation was restricted to the seven studies with clinically confirmed acute events, the overall pattern was unchanged: findings remained inconsistent, with both significant and null associations reported across serological and microbial detection methods. The direction and inconsistency of the evidence were therefore not driven by studies using less rigorous AMI ascertainment; nonetheless, this heterogeneity in outcome definition remains an important source of clinical heterogeneity and is acknowledged as a limitation.

## 4. Discussion

### 4.1. Summary of Main Findings

This systematic review is, to our knowledge, among the first to evaluate the association between *Pg* and AMI specifically, integrating both direct microbial detection and serological evidence within a single pathogen-focused synthesis. Across both approaches, findings were inconsistent: three of six studies in each category reported a significant *Pg*–AMI association, while three in each did not. Discordance persisted even among studies achieving the highest NOS scores, suggesting that inconsistency reflects genuine biological and methodological heterogeneity.

A notable pattern was that associations were most consistent when analyses incorporated the broader subgingival microbiome rather than *Pg* alone. Studies examining combined antibody responses to multiple red complex pathogens [34], total subgingival bacterial burden [38], or the co-presence of several periodontitis-associated bacteria [31] tended to show stronger links to AMI risk. This supports the polymicrobial model of periodontitis and raises the possibility that *Pg* contributes to cardiovascular risk synergistically with co-infecting organisms rather than as a sole driver [17].

From a clinical standpoint, these findings do not currently support using *Pg* detection or anti-*Pg* antibody titres as a stand-alone biomarker for AMI risk prediction or stratification in practice. Even where statistically significant associations were reported, effect sizes were generally modest, derived from case-control designs that cannot establish temporality, and inconsistent across studies using comparable methods. Any clinical significance of *Pg* exposure for AMI risk should therefore be considered alongside, rather than in place of, established cardiovascular risk factors, and any translation into risk-stratification tools or treatment recommendations should await confirmation in prospective, adequately confounder-adjusted studies.

### 4.2. Virulence Mechanisms and Cardiovascular Pathobiology

*Pg* possesses an extensive arsenal of virulence factors—including capsule, fimbriae, gingipains (arginine-specific and lysine-specific cysteine proteases), outer membrane vesicles, and atypical lipopolysaccharides—that collectively enable immune evasion and systemic dissemination [13,14]. Bacteraemia following periodontal manipulation enables *Pg* to invade endothelial cells, smooth muscle cells, and macrophages, perpetuating chronic intravascular inflammation [42]. The detection of *Pg* DNA within atherosclerotic plaques supports a role in direct plaque destabilisation [43], consistent with Wu et al. [41], who linked circulating *Pg* DNA to CAD severity. Additional mechanistic pathways include the *Pg*-induced impairment of cardiomyocyte autophagy [44] and the promotion of macrophage foam cell formation through oxidised low-density lipoprotein uptake [45,46]. Gingipains further contribute to complement subversion, helping *Pg* evade macrophage-mediated phagocytic clearance [47].

### 4.3. Sources of Heterogeneity

Several distinct sources of heterogeneity account for inconsistency across included studies. First, antigen selection for ELISA varied considerably: some studies targeted *Pg* gingipain [32,36], others used a single reference strain [34,35], and others employed multiple *Pg* serotypes [12,37]. Second, only two of the six serological studies quantified IgA in addition to IgG [12,37], and the two isotypes yielded divergent findings in both, highlighting the importance of isotype-resolved immunological analysis. Evidence that IgG2 responses to *Pg* gingipains correlate positively with periodontal pocket depth while IgG4 correlates negatively further underscores this complexity [48].

Third, the number of subgingival sampling sites per participant ranged from 3 to 12, and sampling criteria were not standardised. Studies employing oral swabs rather than subgingival plaque sampling were less likely to identify significant associations [40], confirming that sampling site and depth are critical variables. Fourth, confounder adjustment varied from minimal (age and sex only) to comprehensive, limiting the comparability of adjusted effect estimates across studies.

### 4.4. Comparison with Prior Literature

Prior meta-analyses on periodontitis and cardiovascular outcomes have reported modest but significant associations [25,26], though none have examined *Pg* specifically as the exposure of interest. Joshi et al. [24] provided the nearest precedent, reporting a pooled odds ratio of 1.23 (95% CI 1.09–1.38) for anti-*Pg* IgG in coronary artery disease patients across 20 studies; however, the inclusion of heterogeneous cardiovascular disease subtypes limits direct comparison. The present review contributes a more granular, *Pg*-specific analysis, revealing that even when the same organism is targeted, the direction and magnitude of association vary substantially depending on the exposure metric and detection platform.

### 4.5. Periodontitis as a Confounder, Mediator, or Effect Modifier

A conceptual issue central to interpreting this body of evidence is the relationship between periodontitis and *Pg* exposure itself. Periodontitis could plausibly act as a confounder (a shared cause of both *Pg* exposure and AMI risk through systemic inflammation), a mediator (if *Pg* contributes to AMI risk by inducing periodontal tissue destruction and the resulting systemic inflammation), or an effect modifier (if the *Pg*–AMI association differs according to periodontal disease severity). These three roles imply different, and partly incompatible, analytic strategies. Treating periodontitis solely as a confounder and adjusting for it risks over-adjustment and the attenuation of a true mediated effect, whereas failing to account for it risks residual confounding. Among the included studies, adjustment for periodontal disease status was inconsistent. Periodontal status was assessed in only eight of the 12 studies (Table 1), and none explicitly modelled periodontitis as a mediator or effect modifier of the *Pg*–AMI relationship. The stronger associations observed when analyses incorporated the broader periodontal microbiome or combined antibody panels (Section 4.1) are compatible with a model in which *Pg* acts partly through, rather than independently of, periodontitis, although the current evidence base cannot distinguish between these possibilities. Future studies should explicitly pre-specify whether periodontitis is being modelled as a confounder, mediator, or effect modifier and select statistical approaches, such as mediation analysis, accordingly.

### 4.6. Certainty of the Available Evidence

The overall certainty of the evidence underlying this review is low. All 12 included studies were case-control in design, a design inherently vulnerable to reverse causation and selection bias. Although NOS ratings indicated generally sound methodological quality within individual studies (eight rated good and four rated fair), they cannot overcome the inherent limitations of the case-control design in establishing temporality. As noted in Section 2.6, a formal GRADE assessment was not undertaken given the heterogeneity in outcomes and exposure metrics across studies, so certainty could not be graded across the evidence base as a whole. Accordingly, the association between *Pg* and AMI reported here should be regarded as hypothesis-generating rather than as evidence sufficient to guide clinical practice, and statements in this review regarding the existence, direction, or strength of a *Pg*–AMI association should be read as provisional, subject to revision by higher-certainty prospective evidence.

### 4.7. Strengths and Limitations

Strengths include comprehensive five-database searching with a prospectively registered protocol, restriction of the eligible population to myocardial infarction rather than the broader coronary artery disease spectrum (acknowledging that the method of AMI ascertainment varied across the included studies; see Table 4 and Section 3.5), focus on a single keystone periodontal pathogen, high inter-rater reliability in study selection (Cohen’s kappa = 0.82), and adherence to PRISMA 2020 reporting standards.

Limitations include: (1) all included studies were case-control in design, precluding causal inference; (2) no formal publication bias assessment was conducted—null findings may be under-represented in the literature; (3) GRADE certainty-of-evidence grading was not applied; (4) meta-analysis was not feasible due to clinical and methodological heterogeneity; (5) the exclusion of grey literature and non-English publications may introduce selection bias; and (6) heterogeneity in the ascertainment of AMI across the included studies—ranging from biomarker-, ECG-, and angiography-confirmed acute events to self-reported or previously documented myocardial infarction—limits the comparability of the assembled evidence base.

## 5. Conclusions

This systematic review is, to our knowledge, among the first to evaluate the association between *Porphyromonas gingivalis* and acute myocardial infarction specifically, integrating both direct microbial detection and serological evidence within a single pathogen-focused synthesis. Current evidence from 12 case-control studies is insufficient to support a consistent, independent association between *Pg* and AMI. Findings varied substantially across studies depending on detection platform, antigen selection, anatomical sampling site, immunoglobulin isotype, and degree of confounder adjustment. Stronger and more consistent associations were observed in studies incorporating the broader periodontal microbiome, underscoring the polymicrobial character of periodontitis-associated cardiovascular risk.

Methodological standardisation is urgently needed, including consensus on *Pg* antigen selection, standardised subgingival sampling protocols, immunoglobulin subclass resolution, and comprehensive confounder sets. Prospective cohort studies and, where feasible, interventional trials examining the effect of periodontal treatment on AMI incidence are needed. Comprehensive periodontal assessment and management may contribute to overall systemic health and should be encouraged as part of routine preventive care; however, current evidence does not support the use of *Pg* detection, in isolation, as a validated tool for AMI risk stratification, nor does it establish a causal relationship between *Pg* and AMI.

## Figures and Tables

**Table 1:** Characteristics of the Studies Included in the Review.

Study	Year	Population	*n*	Cases (*n*)	Controls (*n*)	Age Mean (SD)	Male (%)	*Pg* Antigen/Source	Detection Method
(A) Serological Studies-Serum Antibody Analysis									
de Vries et al. [32]	2022	MI/controls with or without PD	1498	779	719	63.2 (6.8)	81.3	*Pg* Rgp gingipain	Serum ELISA/IgG
Holmlund et al. [12]	2011	MI/controls; PD assessed	201	100	101	57.5 (5.3)	79.6	*Pg* multi-serotypes	Serum ELISA/IgA and IgG
Lund Haheim et al. [34]	2008	MI/controls; PD not assessed	1173	548	625	70.8 (4.6)	100.0	ATCC 3277	Serum ELISA/IgG
Lund Haheim et al. [35]	2020	MI/controls; PD not assessed	1172	548	624	70.8 (4.6)	100.0	ATCC 3277	Serum ELISA/IgG
Lysek et al. [36]	2018	MI/controls; PD assessed	210	97	113	60.4 (8.7)	76.7	*Pg* gingipain	Serum ELISA/IgG
Pussinen et al. [37]	2004	MI/controls; PD not assessed	126	63	63	47.6 (6.9)	100.0	*Pg* multi-serotypes	Serum ELISA/IgA and IgG
(B) Microbial Detection Studies-Plaque and Blood Samples									
Andriankaja et al. [31]	2011	Non-fatal MI/controls; PD not assessed	1060	313	747	54.8 (9.6)	53.1	Subgingival plaque (12 sites)	Indirect immunofluorescence
Dogan et al. [33]	2005	GCP with and without AMI; PD assessed	22	11	11	51.9 (6.7)	90.9	Subgingival plaque (6 sites)	Bacterial culture and PCR
Pasupuleti et al. [38]	2013	Acute MI with and without GCP; PD assessed	50	40	10	NR	NR	Subgingival plaque	DNA hybridisation
Seoane et al. [39]	2022	MI/controls with or without PD Stage III/IV	243	131	112	59.5 (8.3)	100.0	Subgingival plaque (3 sites)	PCR
Stein et al. [40]	2009	Acute MI/controls; PD assessed	104	54	50	51.2 (6.4)	93.3	Subgingival plaque (4 deepest sites)	Dot-blot DNA hybridisation
Wu et al. [41]	2023	Acute MI/controls; PD not assessed	460	382	78	58.1 (10.0)	70.0	Oral swab and blood	PCR; cfDNA; gDNA

Note. PD = periodontal disease; *PgPg* = *Porphyromonas gingivalis*; MI = myocardial infarction; AMI = acute myocardial infarction; GCP = generalised chronic periodontitis; ELISA = enzyme-linked immunosorbent assay; PCR = polymerase chain reaction; IgA = immunoglobulin A; IgG = immunoglobulin G; cfDNA = circulating cell-free DNA; gDNA = genomic DNA; NR = not reported; SD = standard deviation.

**Table 2:** Methodological quality assessment using the Newcastle–Ottawa Scale.

Study	Year	Selection (0–4)	Comparability (0–2)	Exposure (0–3)	Total (0–9)	Quality
Andriankaja et al. [31]	2011	3	2	2	7	Good
de Vries et al. [32]	2022	4	2	3	9	Good
Dogan et al. [33]	2005	3	1	2	6	Fair
Holmlund et al. [12]	2011	4	2	3	9	Good
Lund Haheim et al. [34]	2008	3	1	2	6	Fair
Lund Haheim et al. [35]	2020	3	1	2	6	Fair
Lysek et al. [36]	2018	4	1	3	8	Good
Pasupuleti et al. [38]	2013	3	2	3	8	Good
Pussinen et al. [37]	2004	4	2	3	9	Good
Seoane et al. [39]	2022	2	2	2	6	Fair
Stein et al. [40]	2009	3	2	2	7	Good
Wu et al. [41]	2023	4	2	2	8	Good

Note. Three domains evaluated: selection (maximum 4 stars), comparability (maximum 2 stars), exposure (maximum 3 stars). Total maximum = 9. Good quality = 7–9; fair quality = 5–6; poor quality = 0–4.

**Table 3:** Summary of Findings: Association between *Porphyromonas gingivalis* and acute myocardial infarction in included studies.

Study	NOS	Detection Method	Key *Pg* Measure	Main Finding	AMI Association	Confounders Adjusted
de Vries et al. [32]	9	Serum ELISA (IgG)	Anti-Rgp IgG vs. MI/controls	Higher in MI overall (*p* = 0.035); non-significant in PD-only subgroup	Partial	Age, sex, smoking, PD
Holmlund et al. [12]	9	Serum ELISA (IgA, IgG)	Anti-*Pg* IgG and IgA	Elevated IgG in MI (*p* = 0.043); IgA comparable	Yes-IgG	Age, sex, smoking
Pussinen et al. [37]	9	Serum ELISA (IgA, IgG)	Anti-*Pg* IgA and IgG	IgA higher in MI (*p* = 0.035); IgG comparable	Yes-IgA	Age, sex, smoking, diabetes
Lysek et al. [36]	8	Serum ELISA (IgG)	Anti-*Pg* gingipain IgG	Median IgG comparable; moderate titre OR = 2.82 (95% CI 1.02–7.84)	Partial-subgroup	Age, sex, PD status
Lund Haheim et al. [34]	6	Serum ELISA (IgG)	*Pg* IgG; multi-pathogen panel	*Pg* alone non-significant; combined 4-pathogen panel associated with MI risk	No (*Pg* alone)	Cardiovascular risk factors
Lund Haheim et al. [35]	6	Serum ELISA (IgG)	*Pg* IgG; 12.5-year mortality	*Pg* alone non-significant; low anti-T. forsythia predicted CAD mortality	No	CV risk factors; mortality
Stein et al. [40]	7	Dot-blot DNA hybridisation	*Pg* in subgingival plaque	*Pg* independent predictor of AMI (OR = 13.6, 95% CI 3.1–59.8, *p* = 0.0005)	Yes-strongest in review	Age, sex, smoking, BMI, hypertension, statins, cholesterol/HDL
Wu et al. [41]	8	PCR; cfDNA; gDNA (blood)	Oral *Pg*; circulating *Pg* DNA	Oral swab non-significant; blood cfDNA and gDNA higher in AMI (*p* < 0.05)	Partial-blood only	Age, sex, CAD severity
Seoane et al. [39]	6	PCR (subgingival, 3 sites)	*Pg* detection vs. MI/controls	*Pg* driven by PD status; no independent AMI association	No	PD status; CV risk factors
Andriankaja et al. [31]	7	Immunofluorescence (12 sites)	*Pg* prevalence vs. non-fatal MI/controls	*Pg* comparable (18.5% vs. 15.8%, *p* > 0.05); no association in adjusted model	No	Age, sex, smoking, diabetes, BMI
Pasupuleti et al. [38]	8	DNA hybridisation (subgingival)	*Pg* load in MI with vs. without GCP	Higher *Pg* in MI with GCP vs. MI alone (*p* = 0.043)	Partial-via PD	PD status
Dogan et al. [33]	6	Bacterial culture and PCR	*Pg* proportion in GCP with vs. without AMI	Lower *Pg* in GCP with AMI vs. GCP alone (*p* = 0.05)	No-inverse	PD severity

Note. NOS = Newcastle–Ottawa Scale; *Pg* = *Porphyromonas gingivalis*; AMI = acute myocardial infarction; MI = myocardial infarction; ELISA = enzyme-linked immunosorbent assay; PCR = polymerase chain reaction; cfDNA = circulating cell-free DNA; gDNA = genomic DNA; PD = periodontal disease; GCP = generalised chronic periodontitis; OR = odds ratio; CI = confidence interval; CAD = coronary artery disease; HDL = high-density lipoprotein; BMI = body mass index; CV = cardiovascular. Reference numbers are given in square brackets.

**Table 4:** Acute myocardial infarction (AMI) diagnostic criteria used in each included study.

Study	Year	Outcome Label (Table 1)	AMI/MI Diagnostic Criteria	Comments Relevant to Eligibility
Andriankaja et al. [31]	2011	Non-fatal MI/controls	WHO criteria: ≥2 of chest pain, CK-MB elevation, or ECG changes; hospital records (ICD-9 code 410) used for confirmation.	Partial. Included patients with confirmed non-fatal acute myocardial infarction using WHO diagnostic criteria. Restricted to survivors of non-fatal events.
de Vries et al. [32]	2022	MI/controls (with or without PD)	First hospitalized MI (PAROKRANK cohort); previous MI excluded.	Yes. Included patients hospitalized with a first myocardial infarction. Previous myocardial infarction was excluded.
Dogan et al. [33]	2005	GCP with and without AMI	Acute MI diagnosed by ECG changes and elevated cardiac enzymes.	Yes. Acute myocardial infarction diagnosed using ECG findings and cardiac enzyme elevation.
Holmlund et al. [12]	2011	MI/controls; PD assessed	Acute MI confirmed by ECG findings plus elevated CK-MB and troponin T.	Yes. Acute myocardial infarction confirmed using ECG findings together with elevated cardiac biomarkers.
Lund Haheim et al. [34]	2008	MI/controls; PD not assessed	Self-reported history of MI obtained by questionnaire; no clinical verification reported.	Partial. Included participants with a self-reported history of myocardial infarction rather than clinically confirmed acute events.
Lund Haheim et al. [35]	2020	MI/controls; PD not assessed (CVD mortality outcome)	Self-reported history of MI at baseline; primary outcome was cardiovascular mortality.	Partial. Baseline myocardial infarction status was self-reported, while the primary outcome was cardiovascular mortality rather than incident acute myocardial infarction.
Lysek et al. [36]	2018	Past MI/controls; PD assessed	Previous MI identified from cardiology medical records; specific diagnostic criteria not reported.	Partial. Included participants with a documented history of previous myocardial infarction. Time from infarction to assessment was not reported.
Pasupuleti et al. [38]	2013	Acute MI with and without GCP	Acute MI diagnosed by ECG findings and elevated cardiac enzymes.	Yes. Acute myocardial infarction diagnosed using ECG findings and cardiac enzyme measurements.
Pussinen et al. [37]	2004	MI/controls; PD not assessed	Incident fatal and non-fatal MI identified through national hospital and death registries.	Partial. Prospective study of future myocardial infarction identified through national registry data, including fatal and non-fatal events.
Seoane et al. [39]	2022	MI/controls with or without PD Stage III/IV	First acute MI confirmed by ECG, high-sensitivity troponin I, and coronary angiography.	Yes. Included first acute myocardial infarction confirmed using clinical evaluation, cardiac biomarkers, ECG findings, and coronary angiography.
Stein et al. [40]	2009	Acute MI/controls; PD assessed	Acute MI diagnosed according to the 2000 ESC/ACC Universal Definition (symptoms, ECG, and troponin).	Yes. Acute myocardial infarction diagnosed according to the ESC/ACC Universal Definition using clinical symptoms, ECG changes, and troponin elevation.
Wu et al. [41]	2023	Acute MI/controls; PD not assessed	Acute MI diagnosed according to the Fourth Universal Definition with ECG, cardiac biomarkers, and coronary angiography.	Yes. Acute myocardial infarction diagnosed according to the Fourth Universal Definition with biomarker, ECG, and angiographic confirmation.

Note. NOS = Newcastle–Ottawa Scale; PD = periodontal disease; GCP = generalised chronic periodontitis; MI = myocardial infarction; AMI = acute myocardial infarction; CVD = cardiovascular disease.

## Data Availability

No new data were created or analyzed in this study. Data sharing is not applicable to this article.

## Supplementary Materials

The following supporting information can be downloaded at: https://www.mdpi.com/article/10.3390/jcm15145689/s1, Table S1: PRISMA 2020 Checklist; Table S2: Full Database Search Strategies.
